# Insecure attachment and problematic social media use: the chain mediating role of social support and social anxiety

**DOI:** 10.3389/fpsyg.2026.1764295

**Published:** 2026-02-18

**Authors:** Miaomiao Zeng, Liangrong Huang

**Affiliations:** 1 School of Education, Zhaoqing University, Zhaoqing, China; 2 School of Philosophy and Sociology, Jilin University, Changchun, China

**Keywords:** attachment anxiety, attachment avoidance, insecure attachment, problematic social media use, social anxiety, social support

## Abstract

**Background:**

The prevalence of problematic social media use among college students has reached levels that constitute a public health concern. This study aimed to investigate the relationship between insecure attachment (attachment anxiety and attachment avoidance) and problematic social media use, while exploring the potential mediating roles of social support and social anxiety.

**Methods:**

The study utilized the Intimacy Experience Scale, the Interaction Anxiety Scale, the Social Support Scale, and the Problematic Social Media Use Scale to investigate 454 college (*M*_age_ = 20.33, SD = 2.15, 74.2% female).

**Results:**

The results showed that both attachment anxiety and attachment avoidance were positively correlated with problematic social media use. The results of the chain mediation model indicated that both social support and social anxiety independently mediated the relationship between attachment anxiety and problematic social media use. Furthermore, social support and social anxiety acted as chain mediators between attachment anxiety and problematic social media use. A similar chain mediation pathway was identified for attachment avoidance, whereby social support and social anxiety also served as sequential mediators.

**Conclusion:**

These findings offer new insights for the intervention and treatment of problematic social media use. The implications of the results are discussed.

## Introduction

Social media is prevalent all over the world. Social media users (e.g., Facebook, Instagram, TikTok, and WeChat) have grown exponentially, with more than 4 billion active social media users worldwide (She et al., 2023). From 2008 to 2019, the number of users of social media such as Facebook increased from 69 million to 2.45 billion (Ruggieri et al., 2020). While social media serves as an essential tool for daily life and social interaction, its convenience and engaging nature can also facilitate compulsive use, potentially leading to addictive behaviors. Problematic social media use is defined as excessive attention to social media and results from an uncontrollable urge to log on and use social media. This leads to devoting a significant amount of time and energy to social media, interfering with people’s learning, productivity, real-life relationships, development, and mental health (Andreassen and Pallesen, 2014). Problematic social media use is associated with numerous adverse outcomes, including lower life satisfaction, impaired academic and job performance, unhealthy relationships, poor sleep quality, negative affective states such as anxiety and depression, and an increased risk of physical discomfort like neck pain (Andreassen et al., 2014; Rajesh and Rangaiah, 2022; Salari et al., 2023; Wolniczak et al., 2013). Notably, research suggests shared neural mechanisms between problematic social media use and substance use disorders. The prevalence of problematic social media use is considerable, particularly among university students. A meta-analysis reported a global pooled prevalence of 18.4% among 35,520 students, with the rate among Asian students being even higher at 22.8% (She et al., 2023). In particular, a systematic review showed that the prevalence of problematic social media use among Chinese students was 34% (Andreassen, 2015), suggesting that problematic social media use in college populations has evolved into a serious public health concern. Given that the treatment goal for problematic social media use typically cannot be complete abstinence from social media, identifying its risk and protective factors in college students is of paramount importance. Addiction has been conceptualized as an attachment disorder, representing a maladaptive behavior in early adulthood (Höfler and Kooyman, 1996). Correspondingly, individual attachment styles have been identified as significant personality predictors of problematic social media use among college students (Flynn et al., 2018). Social media use can serve an important social compensation function, allowing individuals to manage relationships and seek interpersonal attachment (Kwon et al., 2016). For college students with insecure attachment styles, excessive social media use may represent an attempt to fulfill unmet attachment needs, such as compensating for emotional deficits (Arikan et al., 2022). Therefore, this study examines the relationship between insecure attachment and problematic social media use among college students, as well as to investigate the underlying psychological mechanisms involved.

### Insecure attachment and problematic social media use

Attachment theory posits that early attachment experiences and parent–child interactions shape an individual’s internal working models of self and others. These mental representations subsequently influence psychological organization and affect emotional, cognitive, and behavioral responses in relationships (Ainsworth and Bowlby, 1991). Adult attachment theory extends this framework from parent–child bonds to intimate relationships in adulthood (Hazan and Shaver, 2017; Mikulincer and Shaver, 2010). Insecure attachment in adults comprises two primary dimensions: attachment anxiety and attachment avoidance (Brennan et al., 1998). Attachment anxiety is characterized by an intense desire for closeness coupled with a fear of separation from attachment figures. It reflects a chronic preoccupation with rejection and abandonment, driving a persistent pursuit of emotional intimacy and social connection. In contrast, attachment avoidance involves discomfort with intimacy and dependence on others, leading individuals to distance themselves from close interpersonal connections (Read et al., 2018; Shaver and Mikulincer, 2002). Attachment avoidance reflects the extent to which individuals reject dependence on others and distrust others. Individuals with an avoidant attachment style typically prioritize independence and may experience discomfort with intimate relationships and emotional closeness. A highly anxious attachment style reflects a hyperactivated attachment system, in which individuals seek excessive reassurance and proximity to fulfill attachment needs. Conversely, a highly avoidant attachment style reflects a deactivated attachment system, often suppressing emotional needs and avoiding the formation of close relationships (Liu and Ma, 2019b).

According to attachment theory (Bowlby, 1973), individuals who experience difficulty forming secure intimate relationships may enter a negative attachment state, triggering compensatory behaviors (Keefer et al., 2012). Those with insecure attachments may attempt to compensate for unmet attachment needs through material or behavioral strategies. According to the cognitive-behavioral model, insecure attachment is viewed as a reliable predictor of pathological social media use (Davis, 2001). Thus, a strong theoretical link exists between insecure attachment and problematic social media use. However, empirical findings regarding the distinct roles of attachment anxiety and avoidance in predicting problematic social media use remain inconsistent. While some studies report a positive association between attachment anxiety and problematic social media use (Liu and Ma, 2019b), findings for attachment avoidance are mixed, showing either negative or positive relationships (Blackwell et al., 2017; Worsley et al., 2018). These discrepancies may be attributable to differences in measurement tools, cultural context, or sample characteristics. Therefore, this study will examine the relationship between Insecure attachment, and problematic social media use based on Chinese college students. We hypothesize that both attachment anxiety and attachment avoidance are positively related to problematic social media use among Chinese college students.

### The mediating role of social anxiety

To date, no study has explicitly examined the potential mediating role of social anxiety in the relationship between insecure attachment and problematic social media use. Social anxiety is a common negative emotion among the college student population, and it is a feeling of anxiety that arises from facing or thinking about interpersonal evaluations in social situations (Bowles, 2017; Vassilopoulos et al., 2017). Social anxiety is a risk factor for problematic social media use. Davis’ cognitive behavior model posits psychopathological factors (e.g., social anxiety) are distal necessary precursors to specific pathological internet use behaviors (e.g., problematic social media use) (Davis, 2001). Extending this view, researchers have proposed a social skills model that emphasizes the role of psychosocial deficits, suggesting that socially anxious or deficient individuals are more inclined to socialize online and may overuse social media to compensate for unmet social needs (Caplan, 2003). Similarly, the Interaction of Person–Affect–Cognition–Execution (I-PACE) model (Brand et al., 2019; Brand et al., 2016) proposes that an individual’s core characteristics (including psychopathological and physiological factors), affect, cognition, and executive functions interact to influence specific internet-use behaviors. Within this framework, psychopathological factors like social anxiety are significant contributors to problematic social media use. Individuals with high social anxiety often fear interaction and engagement with others, exhibiting social withdrawal behaviors that can, in turn, fuel urges and cravings for social media use (Naidu et al., 2023). Empirical research supports a positive correlation between social anxiety and problematic social media use (Kim and Bae, 2022).

Based on attachment theory, internal working models guide how individuals perceive and interpret social situations. Insecure attachment may lead to hypervigilance toward signals of rejection or threat in social contexts, fostering cognitive biases related to social threat (Manning et al., 2017). Thus, insecure attachment is closely associated with social anxiety. Vertue (2003) proposed an integrative theoretical model that synthesizes evolutionary, self-presentation, and learning theories of social anxiety from an attachment perspective, positing attachment as a key developmental factor for social anxiety (Vertue, 2003). Vertue (2003) hypothesized that early attachment experiences may lead to negative internal working models (e.g., low self-esteem, shyness) and negative models of others, as well as rejection of other models. These maladaptive models promote evolved avoidance behaviors, which induce and reinforce anxiety in social situations (Brumariu et al., 2013; Ollendick and Benoit, 2012). The integrative theory conceptually complements the cognitive model of social anxiety (Clark and Wells, 1995), which argues that underlying dysfunctional schemas about the self and others predispose individuals to hypervigilance in social settings, perceive them as dangerous, and consequently engage in avoidance behaviors. Empirical evidence confirms a positive correlation between insecure attachment and social anxiety (Read et al., 2018). In summary, based on the theoretical foundations and empirical evidence reviewed, we analyzed the relationships among insecure attachment, social anxiety, and problematic social media use. This study aiming to inform the construction of a pathological model of problematic social media use that incorporates the personality difference perspective of insecure attachment and the mediating mechanism of psychopathological factors. Therefore, we hypothesize that social anxiety mediates the relationship between insecure attachment (i.e., attachment anxiety and attachment avoidance) and problematic social media use among Chinese college students.

### The mediating role of social support

Social support is defined as an individual’s perception or experience of being loved, cared for, respected, and valued within a social network characterized by mutual obligation and assistance (Eskandari and Baratzadeh Ghahramanloo, 2020; Vedder et al., 2005). It can also be described as the assistance available from others when coping with stressful events, which positively influences the resolution of maladaptive behaviors (Bilgin and Taş, 2018). Attachment style significantly influences the perception and utilization of social support. Individuals with anxious attachment often report greater needs for support yet perceive less support available to them. Furthermore, even when support is received, adults high in attachment anxiety tend to express lower satisfaction with it (Ognibene and Collins, 1998). Some scholars suggest that both anxiously and avoidantly attached individuals may be prone to encoding and recalling helping behaviors as less supportive (Moreira et al., 2003). Those with insecure attachments are generally more reluctant to trust others, less inclined to seek support, and consequently receive lower levels of social support (Zerach and Elklit, 2020). Consistent with this view, numerous empirical studies indicate that both attachment anxiety and avoidance are negatively correlated with perceived social support (Florian et al., 1995).

According to the cognitive-behavioral model, a lack of social support is a key risk factor for pathological addictive behaviors, with social isolation predisposing individuals to problematic social media use (Davis, 2001; Green et al., 2024). This deficiency may drive individuals to seek connection and increase their engagement in social media and online spaces (Eskandari and Baratzadeh Ghahramanloo, 2020). Social compensation theory similarly posits that when individuals do not receive sufficient offline social support, they may attempt to compensate for these unmet needs through social media, often leading to overuse (Bonetti et al., 2010). Empirical evidence supports a negative correlation between social support and problematic social media use (Huang et al., 2026). Roberts et al. (1996) proposed that social support may mediate the relationship between adult attachment anxiety and psychological problems or maladaptive behaviors, a proposition supported by subsequent empirical work. This perspective aligns with the social support buffer model (Cohen and Wills, 1985), which posits that social support can mitigate the negative effects of insecure attachment, thereby potentially reducing the likelihood of problematic social media use. Therefore, we hypothesized that social support mediates the relationship between insecure attachment and problematic social media use among Chinese college students.

### The chain-mediated role of social support and social anxiety

The relationship between social support and social anxiety is also well established, and both factors are recognized as significant interpersonal influences on problematic social media use. According to the social causation model, a lack of social support can contribute to the development of social anxiety (Panayiotou and Karekla, 2013). Individuals experiencing deficient social support may be more likely to perceive others’ behavior in social situations as threatening, increasing their vulnerability to social anxiety and subsequent social avoidance (Brissette et al., 2002). Empirical research supports this connection, demonstrating a negative correlation between social support and social anxiety, wherein lower perceived social support is associated with higher levels of social anxiety (Moghtader and Shamloo, 2019). Therefore, we hypothesize that social support and social anxiety sequentially mediate the relationship between insecure attachment and problematic social media use among Chinese college students.

## Methods

### Participants

A total of 454 subjects were from two public universities in southern China (convenience sampling). Subjects participated in this study voluntarily, and their age range was between 18 and 30 years old (*M*_age_ = 20.3 SD = 2.15, female = 337). Among them, 352 are undergraduate students and 102 are graduate students.

### Measurements

#### Insecure attachment

The Experiences in Close Relationships (ECR) scale was used to assess insecure attachment in adults. The ECR was developed by Brennan et al. (1998) and others based on previous research (Brennan et al., 1998). Brennan collected items from a large number of previously developed scales and conducted a large-scale test, which resulted in a factor analysis of 36 items, half of which reflected the attachment avoidance dimension and the other half reflected the attachment anxiety dimension. Each item on the scale has a scale of 1–7, and subjects choose according to their feelings, with higher scores on the dimensions indicating higher levels of attachment anxiety or avoidance. In this study, Cronbach’s alpha for the Attachment Avoidance Scale was 0.84 and Cronbach’s alpha for the Attachment Anxiety Scale was 0.87. The scale has been widely used both at home and abroad. This scale has been widely used at home and abroad.

#### Social anxiety

The interaction Anxiousness Scale (IAS) was originally developed by Leary in 1983 using a clinical empirical method, and the Chinese version was revised by Chunzi Peng, Liangshi Yan, Xiaohong Ma, and Wenli Wu in 2004. The scale is designed to measure subjective anxiety about interpersonal interactions and consists of 15 self-reported items. The scale is scored on a 5-point scale (from “1” to “5” representing “not at all” to “completely”). In the present study, Cronbach’s alpha for this scale was 0.84.

#### Social support

The Social Support Rating Scale (SSRS) developed by Xiao Shuiyuan was used to evaluate the social support of college students. The scale was divided into three dimensions, with a total of 10 entries: subjective support dimension (4 entries); objective support dimension (3 entries); and support utilization dimension (3 entries). The scale is widely used, with Cronbach’s alpha for previous studies ranging from 0.62 to 0.77 (Cui and Chi, 2021; Hu et al., 2023; Zou et al., 2023). In the present study, Cronbach’s alpha for this scale was 0.66.

#### Problematic social media use

The Bergen Social Media Addiction Scale (BSMA), which was developed by Andreassen and Pallesen (2017) (Andreassen et al., 2017), is a 6-item scale with 6 dimensions: salience, craving, emotion regulation, relapse, withdrawal, and conflict, which relate to experiences that have occurred in the last experiences that have occurred in the past year and are scored on a 5-point scale (“1-never” to “5-always”). The scale has been widely used. In the present study, Cronbach’s alpha for this scale was 0.83.

### Data analysis

SPSS26.0 and SPSS macro Process3.5 (Hayes, 2009) plug-ins were used for data processing. Data were standardized for all measures in this study; a one-way approach was used to test for the presence of common method bias; Pearson product-difference correlation was used to explore the relationship between the main variables; and a bias-corrected nonparametric percentile Bootstrap method was used to test for mediating effects (Model 6). In our analyses, we used age as a control variable, which has also been used as a control variable in previous related studies (Blackwell et al., 2017).

## Results

### Test for common method bias

In this study, the research data were subjected to a common method bias test. The data were factor analyzed according to the Harman one-way test. The results show that there are 15 factors with an eigenvalue greater than 1 and the percentage of variance of the first principal factor is equal to 15.58% (below the 40% criterion), so it indicates that the common method bias is not serious.

### Descriptive statistics and correlation analysis

According to the results of descriptive statistics and Pearson correlation analysis (see Table 1), attachment anxiety was positively correlated with social anxiety and problematic social media use and negatively correlated with social support. Attachment avoidance was positively associated with social anxiety and problematic social media use and negatively associated with social support.

**Table 1:** Descriptive statistics and correlation analysis.

Variables	*M*	SD	1	2	3	4
1. Attachment anxiety	65.43	18.71	1			
2. Attachment avoidance	58.67	16.09	0.10^*^	1		
3. Social support	37.94	6.01	−0.13^**^	−0.28^**^	1	
4. Social anxiety	46.57	9.71	0.38^**^	0.11^*^	−0.33^**^	1
5. Problematic social media use	14.83	4.98	0.36^**^	0.12^*^	−0.19^**^	0.32^**^

^*^p < 0.05; ^**^p < 0.01.

### Chained mediation model analysis

**Figure 1:**
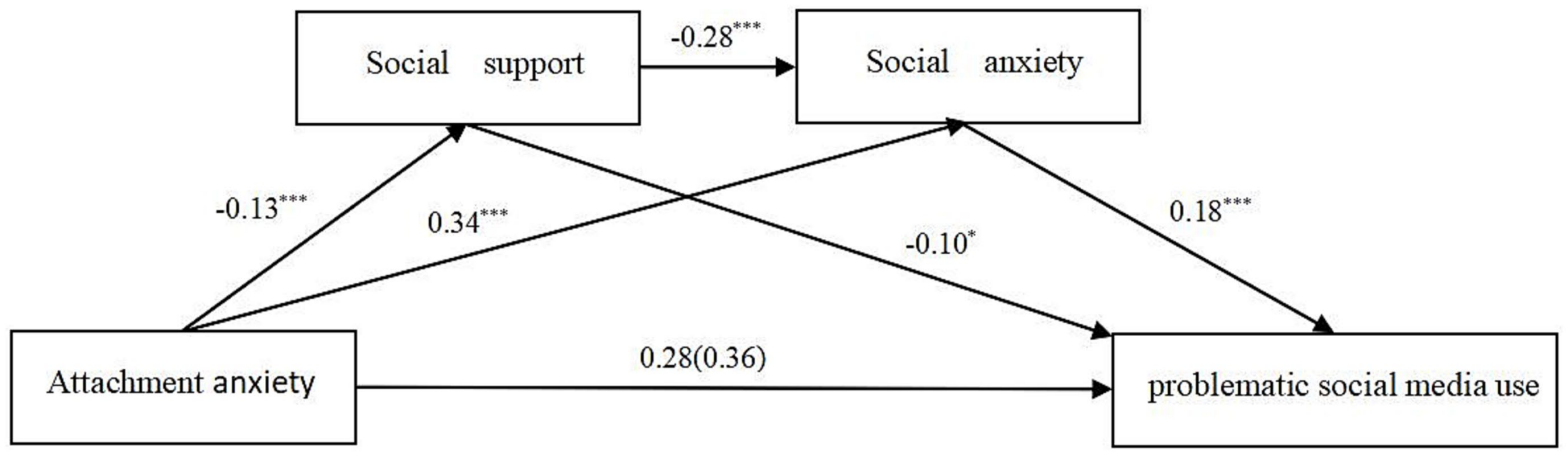
Chain mediation model indicating the mediating roles of social support and social anxiety in the association between attachment anxiety and problematic social media use. ^*^p < 0.05; ^**^p < 0.01; ^***^p < 0.001. Standardized coefficients are reported.

**Figure 2:**
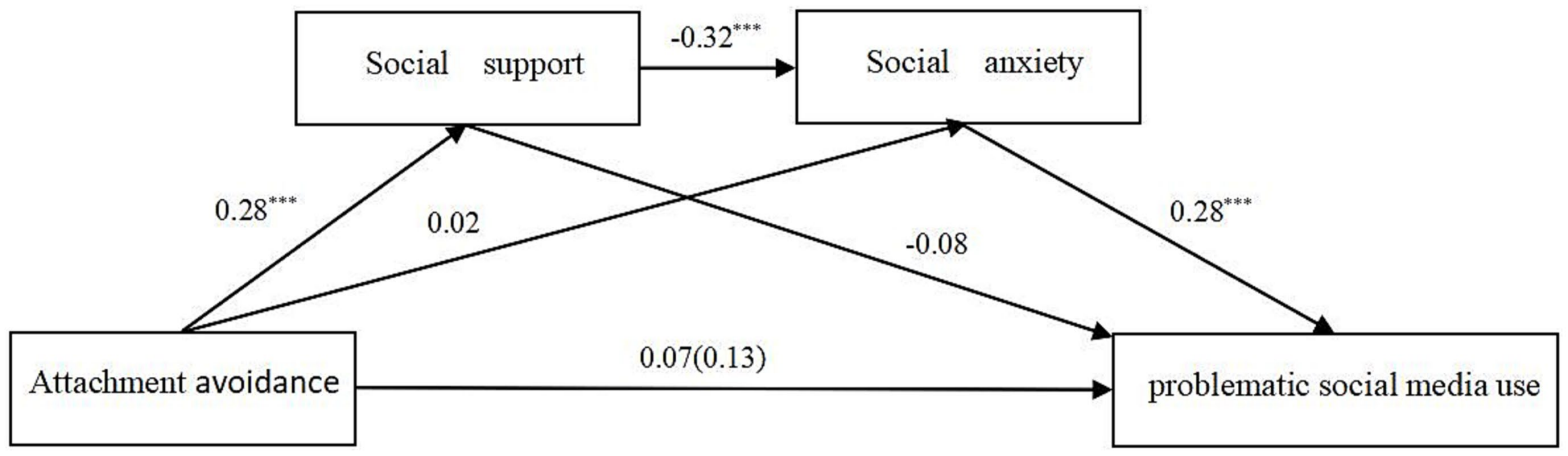
Chain mediation model indicating the mediating roles of social support and social anxiety in the association between attachment avoidance and problematic social media use. ^*^p < 0.05; ^**^p < 0.01; ^***^p < 0.001. Standardized coefficients are reported.

Chain mediation was examined using Model 6 in the SPSS PROCESS program created by Hayes. Age was used as a control variable in the analysis. Results showed that attachment anxiety significantly positively predicted social anxiety and problematic social media use (*β* = 0.34, *p* < 0.001; *β* = 0.28, *p* < 0.001, respectively, see Figure 1), while negatively predicting social support (*β* = −0.13, *p* < 0.001). Social support significantly negatively predicted problematic social media use (*β* = −0.10, *p* < 0.001), and social anxiety significantly positively predicted problematic social media use (*β* = −0.18, *p* < 0.001). The results showed that attachment avoidance significantly negatively predicted social support (*β* = −0.28, *p* < 0.001), while attachment avoidance did not significantly predict social anxiety and problematic social media use (*β* = 0.02, *p* > 0.05; *β* = 0.07, *p* > 0.05, respectively, see Figure 2). The significance of the mediating effects was tested using the Bootstrap method as well as repeated sampling 5,000 times. The results showed that the direct effect of attachment anxiety and problematic social media use, the independent mediating effect of social support and social anxiety, and the chained mediating effect of social support and social anxiety were all significant, and none of the Bootstrap 95% confidence intervals contained 0. The direct effect of attachment anxiety and problematic social media use was 0.283, see Table 2. The mediating effect of social support and social anxiety had three paths with a total indirect effect of 0.081: (1) the media pathway of Attachment anxiety → social support → problematic social media use, with an effect of 0.013; (2) the media pathway of Attachment anxiety → social anxiety → problematic social media use, with an effect of 0.062; (3) the chain mediation pathway of Attachment anxiety → social support → social anxiety → problematic social media use, with an effect of 0.007. In addition, the chain-mediated effects of social support and social anxiety between attachment avoidance and problematic social media use belonged to the full chain-mediated model. Bootstrap None of the 95% confidence intervals contained zero. The chain mediation pathway of Attachment avoidance → social support → social anxiety → problematic social media use, with an effect of 0.025, see Table 3.

**Table 2:** Chain mediating paths between attachment anxiety and problematic social media use.

Mediation model paths	Effect	SE	95%CI	
			Upper	Lower
Total effect	0.365	0.043	0.278	0.451
Direct effect	0.283	0.046	0.193	0.374
Total indirect effect	0.081	0.023	0.038	0.127
Attachment anxiety → social support → problematic social media use	0.013	0.007	0.001	0.029
Attachment anxiety → social anxiety → problematic social media use	0.062	0.019	0.025	0.101
Attachment anxiety → social support → social anxiety → problematic social media use	0.007	0.004	0.001	0.014

Standardized coefficients are reported.

**Table 3:** Chain mediating paths between attachment avoidance and problematic social media use.

Mediation model paths	Effect	SE	95%CI	
			Upper	Lower
Total effect	0.128	0.046	0.036	0.219
Direct effect	0.075	0.046	−0.016	0.165
Total indirect effect	0.053	0.019	0.015	0.093
Attachment avoidance → social support → problematic social media use	0.023	0.012	−0.021	0.033
Attachment avoidance → social anxiety → problematic social media use	0.005	0.014	−0.021	0.034
Attachment avoidance → social support → social anxiety → problematic social media use	0.025	0.007	0.012	0.041

Standardized coefficients are reported.

## Discussion

Previous research has established a robust link between attachment and problematic social media use. However, the specific mechanisms through which insecure attachment leads to problematic social media use remain less clearly understood. Grounded in theoretical frameworks and empirical evidence, the present study proposed and tested a chain mediation model to elucidate this relationship, with a specific focus on the interpersonal dynamics characteristic of college students. For attachment anxiety, findings revealed both the independent mediating effects of social support and social anxiety, as well as their sequential mediating effect. For attachment avoidance, only the sequential mediation through social support and social anxiety was identified.

### The relationship between insecure attachment and problematic social media use

This study investigated the association between insecure attachment and problematic social media use within the context of Chinese college students. The results indicated that both attachment anxiety and attachment avoidance were significantly and positively correlated with problematic social media use. This suggests that individuals scoring high on either attachment anxiety or avoidance may be more vulnerable to developing problematic social media use. Notably, while the correlation between attachment anxiety and problematic social media use was moderate, the correlation involving attachment avoidance was weak, indicating that the latter relationship may be less consistent. These findings align with some previous research (Liu and Ma, 2019b). Individuals with high attachment anxiety are driven to seek support and comfort to alleviate feelings of insecurity. Consequently, they may overuse social media to fulfill several interpersonal goals: (1) promoting positive relationships (Andangsari et al., 2013), (2) mitigating negative self-perceptions (Yaakobi and Goldenberg, 2014), (3) reducing the perceived risk of rejection (Jenkins-Guarnieri et al., 2012), and (4) managing fears of abandonment (Andangsari et al., 2013). However, while social media may transiently boost positive affect, this effect often diminishes during face-to-face interactions (Hart et al., 2015). This discrepancy can reinforce compulsive social media use among anxiously attached individuals, ultimately impairing daily functioning and mental health (Hart et al., 2015). Furthermore, their heightened sensitivity to others’ feedback may intensify their efforts to seek such validation online, making it more difficult to regulate their social media use. Moreover, both individuals with attachment anxiety and those with attachment avoidance may turn to more readily accessible alternatives to compensate for unmet attachment needs. Social media, as a convenient and feature-rich platform, offers an easily accessible means to satisfy psychological needs such as belonging, thereby increasing the risk of problematic social media use.

### The mediating roles of social support and social anxiety

Building on this foundation, the present study found that social anxiety mediates the relationship between attachment anxiety and problematic social media use. This finding validates the pathological model of problematic social media use proposed herein. This suggests that social anxiety may serve as a core vulnerability factor in the development of problematic social media use (Wu et al., 2024). Furthermore, it supports the cognitive-behavioral model, confirming that both predisposing factors (attachment anxiety) and psychopathological factors (social anxiety) are significant risk factors for problematic social media use. The results are also consistent with the integrative model proposed by Vertue. Specifically, individuals with high attachment anxiety develop negative cognitive schemas and internal working models, leading to hypervigilance in social situations. They are prone to perceiving interpersonal cues as threatening, fearing rejection, and consequently developing avoidance behaviors and anxiety. According to attachment theory, early negative attachment experiences foster negative interpersonal schemas and emotional dysregulation, increasing long-term vulnerability to psychopathology such as social anxiety (Goodwin, 2003). Those with high attachment anxiety, operating from a negative internal working model, may anticipate social isolation or rejection, making them more susceptible to social anxiety in daily interactions (Manning et al., 2017). Social media offers a virtual environment where individuals with high social anxiety can conceal physical symptoms of discomfort, such as red faces, breathlessness, and nervousness (Enez Darcin et al., 2016; Kardefelt-Winther, 2014). This platform affords them a greater sense of control over interactions, potentially reducing immediate negativity and fear of critical evaluation. However, this dynamic may also predispose them to problematic social media use (Cheng et al., 2019; Chu et al., 2021; Stritzke et al., 2004). According to theories of computer-mediated communication, social media provides a rich social resource for individuals with social deficits or interpersonal difficulties, allowing them to compensate for unmet needs in face-to-face interactions. Nevertheless, excessive online engagement may ultimately prove insufficient for fulfilling core emotional needs (Meshi et al., 2020), resulting in the gradual development of problematic social media use. Notably, social anxiety did not mediate the relationship between attachment avoidance and problematic social media use. This suggests that attachment avoidance may not predict heightened social anxiety, a finding consistent with some prior research. Avoidantly attached individuals may actively distance themselves from close relationships, thereby circumventing the interpersonal situations that typically provoke anxiety. An alternative explanation may relate to measurement specifics.

This study also found that social support mediates the relationship between attachment anxiety and problematic social media use, indicating its potential role as a protective factor. Higher perceived social support appears to reduce the risk of problematic social media use, while lower support increases it. This result validates the cognitive-behavioral model (Davis, 2001), confirming that predisposing factors (attachment anxiety) coupled with low social support constitute significant risk factors for problematic social media use. When college students struggle to establish an effective offline support system, they may turn to social media to alleviate negative emotions, potentially leading to overuse. This finding also supports Roberts’ proposition that social support mediates the link between attachment anxiety and maladaptive behaviors. Individuals high in attachment anxiety may doubt their relational value and consequently express lower satisfaction with received support (Vogel and Wei, 2005). Although such individuals often perceive receiving less support, they may nonetheless actively seek it (Hart et al., 2015; Zerach and Elklit, 2020). According to the social compensation theory, individuals with low social support may employ compensatory strategies to meet their needs. With its ease of access, social media becomes a likely tool for seeking feedback and connection, thereby increasing the risk of addictive usage patterns (Liu and Ma, 2019b). While supportive interactions can alleviate stress and mitigate the impact of adverse events, the very drive to seek such support online may inadvertently foster problematic social media use (Mao and Zhao, 2023). Conversely, a mediating role for social support was not established between attachment avoidance and problematic social media use. A possible explanation is that although individuals high in avoidance typically receive and report lower satisfaction with social support, they may perceive high support as a threat to their independence. Consequently, they may inhibit support-seeking behaviors and are less likely to use social media to obtain potential support, thus weakening this pathway (Kizuki and Fujiwara, 2018).

According to the social causation model (Panayiotou and Karekla, 2013), low social support can contribute to social anxiety. A lack of support may undermine self-confidence in social situations and heighten sensitivity to potential negative evaluation, eroding positive self-image and increasing social anxiety (Moghtader and Shamloo, 2019). The buffer model further posits that social support mediates the relationship between insecure attachment and subsequent psychological issues (e.g., social anxiety) or maladaptive behaviors (e.g., problematic social media use). Social support is thought to buffer negative affect, reduce adverse emotional experiences, and enhance adaptive coping. Individuals high in attachment anxiety or avoidance, who typically have less access to and satisfaction with social support, may hold lower self-views and exhibit heightened sensitivity to perceived social threats. This can precipitate social anxiety, which they may then struggle to manage in face-to-face settings. Consequently, they may overuse social media to alleviate this anxiety, ultimately leading to problematic social media use.

### Limitation and recommendation

First, the sample of our study mainly included colleges and universities in the southern provinces, which may limit the generalizability of the findings to other geographic regions. Therefore, future research could further increase the sample scope and sample size. Second, our study is a cross-sectional study, which is not able to expose the causal relationship between variables. Therefore, future research could take the approach of examining the causal relationship between variables through longitudinal study. Although Harman’s single-factor test indicated no severe common method bias, this statistical approach has inherent limitations. Future research could employ alternative methods to further examine potential common method bias. The valid sample in this study consisted predominantly of female participants, which may limit the generalizability of the findings to broader populations. Future research should seek a more balanced gender distribution to further validate these results. Finally, the study focused on college students aged 18–30 years. Older adults may differ in their access to social support and susceptibility to social anxiety, which could influence the proposed mediating mechanisms. Extending the age range in future research would help to clarify the roles of social support and social anxiety across different developmental stages.

## Conclusion

This study elucidates the relationship and underlying mechanisms between insecure attachment and problematic social media use among college students. The findings indicate that insecure attachment can influence problematic social media use both directly and indirectly through the mediating roles of social support and social anxiety. Consequently, interventions for problematic social media use should give particular attention to individuals with insecure attachment styles, especially those high in attachment anxiety, given its strong association with problematic social media use in this population. The study contributes a pathological model of problematic social media use in which social anxiety mediates the link between attachment anxiety and problematic social media use, highlighting social anxiety as a core psychopathological factor. Interventions for problematic social media use should therefore specifically address social anxiety symptoms. Furthermore, by incorporating social support into the model, the study expands the current understanding of problematic social media use pathology. On one hand, it clarifies social support as a potential mediator in the developmental pathway. On the other hand, it identifies social support as a protective factor, thereby broadening the scope of interpersonal mechanisms that may help mitigate the risk of problematic social media use among college students. In summary, insecure attachment, social anxiety, and social support are important factors influencing problematic social media use. Future intervention programs and research should consider targeting these psychological and interpersonal dimensions to effectively prevent and reduce problematic social media use.

## Data Availability

The raw data supporting the conclusions of this article will be made available by the authors, without undue reservation.
